# The Potential Role of Platelet-Related microRNAs in the Development of Cardiovascular Events in High-Risk Populations, Including Diabetic Patients: A Review

**DOI:** 10.3389/fendo.2018.00074

**Published:** 2018-03-20

**Authors:** Justyna Pordzik, Katarzyna Pisarz, Salvatore De Rosa, Axel Dyve Jones, Ceren Eyileten, Ciro Indolfi, Lukasz Malek, Marek Postula

**Affiliations:** ^1^Center for Preclinical Research and Technology CEPT, Department of Experimental and Clinical Pharmacology, Medical University of Warsaw, Warsaw, Poland; ^2^Division of Cardiology, Department of Medical and Surgical Sciences, “Magna Graecia” University, Catanzaro, Italy; ^3^URT-CNR, Department of Medicine, Consiglio Nazionale delle Ricerche of IFC, Catanzaro, Italy; ^4^Faculty of Rehabilitation, University of Physical Education, Warsaw, Poland

**Keywords:** biomarker, microRNA, platelet microvesicles, platelet reactivity, cardiovascular diseases, type 2 diabetes mellitus

## Abstract

Platelet activation plays a pivotal role in the development and progression of atherosclerosis, which often leads to potentially fatal ischemic events at later stages of the disease. Platelets and platelet microvesicles (PMVs) contain large amounts of microRNA (miRNA), which contributes largely to the pool of circulating miRNAs. Hence, they represent a promising option for the development of innovative diagnostic biomarkers, that can be specific for the underlying etiology. Circulating miRNAs can be responsible for intracellular communication and may have a biological effect on target cells. As miRNAs associated to both cardiovascular diseases (CVD) and diabetes mellitus can be measured by means of a wide array of techniques, they can be exploited as an innovative class of smart disease biomarkers. In this manuscript, we provide an outline of miRNAs associated with platelet function and reactivity (miR-223, miR-126, miR-197, miR-191, miR-21, miR-150, miR-155, miR-140, miR-96, miR-98) that should be evaluated as novel biomarkers to improve diagnostics and treatment of CVD.

## Introduction

Platelets largely contribute to the progression of atherosclerosis and the development of its clinical complications (1, 2). Upon platelet adhesion to damaged loci of blood vessel walls, at sites of endothelial cell activation, they promote the growth of chronic atherosclerotic plaques, and precipitate the onset of arterial thrombosis following atherosclerotic plaque rupture (3). In addition, platelet activation can induce and maintain a local pro-atherothrombotic mileu, through specific alterations of the arterial wall (4). Platelets are therefore the key cellular component of athero-thrombosis. Notwithstanding their major impact on the development of potentially fatal ischemic events in late phases of cardiovascular diseases (CVD), several aspects of the underlying molecular mechanisms driving platelet activation are yet to be fully clairified (5). Type 2 diabetes mellitus (T2DM) represents a dangerous threat to health worldwide, and up to 50–75% of deaths are due to its macrovascular complications (6, 7). In line with previous reports, platelet reactivity is a critical contributing element to the development of cardiovascular complications in T2DM population (6).

Even though platelets are devoid of nucleus and genomic DNA, they have the capacity to translate inherited messenger RNA (mRNA) into protein. In fact, a strong relationship was demonstrated between transcriptome and proteome in platelets (8, 9). The combination of several distinctive features possibly enables posttranscriptional modulation of gene expression within platelets. Since they are equipped with a complex translational apparatus, as well as unique transcriptome and correlating proteomic profile, they have the capacity to sustain *de novo* translation (10). Interestingly, in recent years it was reported that microRNAs (miRNAs) do not act exclusively on the intracellular level but they also exert their influence extracellularly (11). Exosomes, which represent compact plasma membrane-derived vesicles released by numerous cell types into the extracellular space, function as intercellular signaling molecules (12). They accommodate bioactive proteins, lipids, DNA, mRNAs, and miRNAs carrying significant biological information and deliver them to specific recipient cells (13, 14). Exosomes are able to carry distinct quantities of miRNAs that transfer biological information inbetween cells (12). Upon activation, platelets secrete microvesicles (MVs) containing growth factors and several effector proteins, as well as multivesicular bodies and exosomes, which are able to exert extracellular effects (10, 15). Platelet microvesicles (PMVs) play a role in maintenance of hemostasis, vascular health, and immunity; however, they are also involved in thrombotic and inflammatory disturbances (15). Most importantly, PMVs represent intercellular carriers of Ago2—miRNA complexes, such as miR-223, that exert regulation of gene expression in endothelial cells. This response is considered pro-inflammatory and likely to contribute to the development of cardiovascular events (16).

microRNAs are small, endogenous, noncoding RNAs (17). They regulate a significant proportion of protein coding genes through the interaction with mRNAs (17, 18). This effect is exerted by binding corresponding parts of mRNA transcripts to suppress their translation and control degradation (19).

Until now, 1,881 miRNA sequences have been listed for *Homo sapiens* (20). However, in a recent analysis, less than 20% of human miRNAs met the criteria of high confidence for miRNAs annotation, based on deep sequencing technology (21). Since miRNAs can be easily measured by real-time polymerase chain reactions (PCR) in plasma or serum, they have attracted special interest as potential novel biomarkers and instrument to discover the process of platelet gene expression (22). They provide opportunities to study, observe, as well as control platelet function and vascular condition in patients with potentially higher risk of developing cardiovascular events. Moreover, circulating miRNAs may reflect platelet activation, and therefore, may serve as a substitute marker of efficacy of antiplatelet therapy. However, further studies should be designed to elucidate the mechanism and investigate the associations between miRNA and cardiovascular diseases.

In this systematic review, we present an overview of current knowledge on diagnostic and prognostic value of miRNAs related to platelets in patients with CVD and/or T2DM.

## Article Search Process

**Figure 1:**
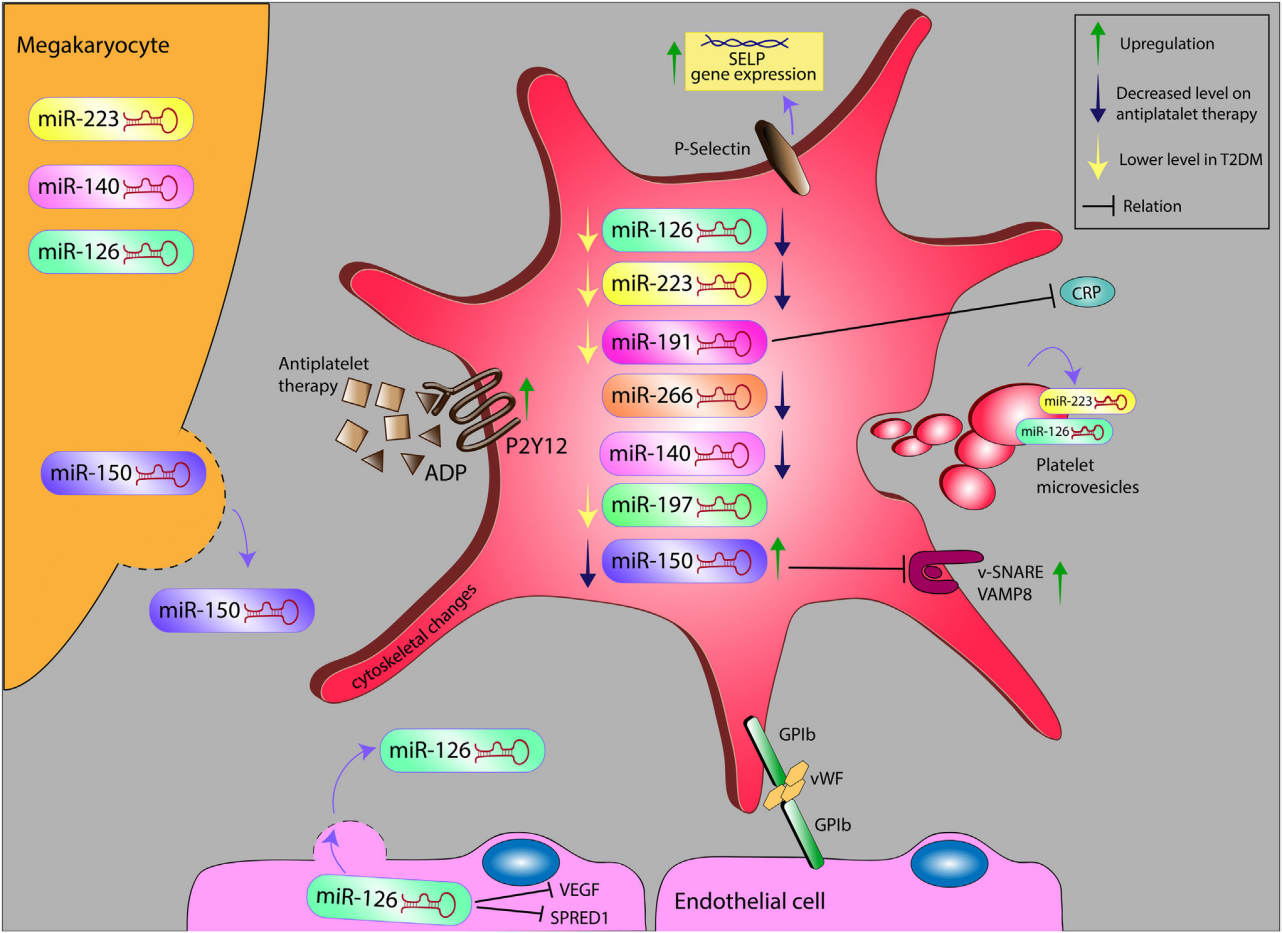
Alteration levels of miR in platelet and platelet microvesicles, and their possible relation with inflammatory markers on antiplatelet therapy in diabetes. miR, microRNA; ADP, adenosine diphosphate; VEGF, vascular endothelial growth factor; SPRED1, sprout-related EVH1 domain-containing protein 1; GP1b, glycoprotein Ib; vWF, von Willebrand factor; SELP, selectin P; VAMP8, vesicle-associated membrane protein 8; CRP, C-reactive protein.

The electronic databases PubMed and Scopus were searched through October 23rd, 2017 for studies that evaluated potential prognostic role of miRNA associated with platelet reactivity in diabetics, using the following search syntax: “Search (“micrornas” [MeSH Terms] OR “mir” [MeSH Terms] OR “mirna” [MeSH Terms]) AND (“platelets” [MeSH Terms] OR “platelet activation” [All Fields] OR “platelet aggregation” [All Fields]) AND (“diabetes mellitus” [MeSH Terms] OR “diabetes” [All Fields]) AND (“prognosis” [MeSH Terms] OR “prognosis” [All Fields]) Filters: Humans. Our search was limited to human studies and did not exclude studies based on ethnicity of study participants. A total of 50 records were identified after duplicates removal. Titles and abstracts were screened by two independent operators, with exclusion of 26 records for any of the following reasons: (a) they were not related to the specific research question (*n* = 6); (b) they did not present original data (*n* = 18); they were not human studies (*n* = 2). Finally, 24 articles were selected to be used in this review. Figure 1 reports the article selection flowchart.

## Most Abundant miRNAs in Platelets

Platelets express high levels of miRNAs. miR-223, miR-126, miR-197, miR-24, and miR-21 represent the most abundant miRNAs in human platelets and PMVs, as shown during microarray screening (10). Moreover, flow cytometry showed that circulating miRNA levels correspond with PMVs level (10). Interestingly, these miRNAs have been reported to correlate with CVD and are emerging as potential biomarkers for risk assessment in CVD and monitoring of antiplatelet drug efficacy (23, 24). Specific miRNA signatures had already been identified to be specifically associated to T2DM and could therefore be exploited as biomarkers of this disease in the future (see Figure 1) (25–31). As novel biomarkers and technologies arrive at the horizon, the use of platelet miRNA testing in CVD and T2DM appears to take on a new aspect.

## miR-223

miR-223, richly expressed in platelets and megakaryocytes, is involved in the development of the hematopoietic lineage (5). The gene encoding miR-223 is on the X chromosome (8). In a landmark study, Landry et al. confirmed that human platelets contain a plentiful array of miRNAs, with miR-223 being one of the most abundant ones (32). Similar evidence was independently reported by another group (33).

Despite the fact that multiple reports have been published on miRNAs thus far, the information on their function in platelets is still scarce. Landry et al. also found that miR-223 targets the adenosine diphosphate (ADP)-receptor P2Y12, a purinergic receptor known to have an impact on platelet reactivity, by amplifying aggregation induced by all known platelet agonists. By identifying the binding site for miR-223 on the P2Y12 mRNA, as well as demonstrating the capacity of miR-223 to control gene expression of platelet precursor cells, they provided a relevant piece of information supporting the hypothesis that P2Y12 expression is regulated by miRNAs in human platelets (8, 32, 34).

Moreover, Nagalla and colleagues revealed that miRNA profiles are linked to, and may predict, the response of platelet aggregation to epinephrine (33). Several studies were then conducted to validate the concept that plasma levels of miR-223 become lower in patients treated with antiplatelet therapy (19, 22, 35, 36). According to Shi et al., platelet miR-223 downregulation correlated with a weaker response to P2Y12 receptor antagonist clopidogrel in a population of 33 Chinese patients (19). The link between circulating miR-223 levels and responsiveness to clopidogrel in patients with coronary heart disease (CHD) was further investigated by Zhang et al. During an analysis of 62 patients with troponin-negative non-ST segment elevation acute coronary syndrome (NSTE-ACS) they found that a decrease in circulating levels of miR-223 was the only independent predictor for platelet reactivity index (PRI)-determined lower responders (OR 0.111, 95% CI: 0.018–0.692, *P* = 0.019), even though it was tested among known predictors of platelet reactivity (e.g., CYP2C19*2/*3 loss-of-function genotypes, use of calcium channel blockers/proton-pump inhibitors, age, diabetes, smoking) (35). On the contrary, results obtained by Chyrchel et al. in 21 males with CHD argued against the hypothesis that plasma levels of miR-223 mark platelet responsiveness to DAPT. More potent platelet inhibition related predominantly to novel P2Y12 antagonists appeared to coexist with higher miR-223 compared to subjects with diminished responsiveness to DAPT (36). Recently, Kaudewitz et al. reported that antiplatelet therapy decreases plasma levels of platelet miRNAs, including miR-223. In a cohort of 125 patients with a previous acute coronary syndrome (ACS), the key platelet-related miR-223 was correlated with platelet function tests (*r*_p_ = 0.28; *n* = 121; *P* = 0.002) (18). The emerging evidence of more effective platelet inhibition, resulting in decrease of miR-223, highlights the potential role of this miRNA in monitoring the process of platelet activation and efficacy of antiplatelet therapy (1).

To date, three studies analyzed the association of miR-223 with clinical endpoints (37–39). Schulte evaluated 873 individuals and revealed that elevated miR-223 levels reliably predicted future cardiovascular deaths [HR 2.23 per one SD increase (1.20; 4.14), *P* = 0.011, C-index 0.80], as 2.1% (*n* = 18) of the subject experienced cardiovascular death over a median follow-up time of 4 years (37). Also Zampetaki and colleagues investigated the link between baseline miRNA quantity and incident myocardial infarction in a population of 820 patients and found that miR-223 was negatively related to disease risk (38). More recently, Keller et al. analyzed a panel of miRNAs to predict outcome in the context of cardiovascular disease prevention. They determined that all-cause 5-year mortality was associated with lower miR-223 levels (HR: 0.30; 95% CI: 0.08–1.07; *P* = 0.063) (39). The presented results show that miR-223 may not only serve as a biomarker of platelet activation, but could also be exploited as a prognostic marker.

Interestingly, lower plasma levels of miR-223 were found in T2DM (25, 34, 40). In particular, miR-223, together with miR-126, miR-140, and miR-26b are expressed at a lower level in both platelets and megakaryocytes from T2DM patients, leading to upregulation of P2Y12 receptor and SELP (P-selectin), thus contributing to platelet hyperactivation (34). In accordance with these findings, a pilot study demonstrated that hyperglycemia-associated downregulation of miR-223 and miR-146a mediates platelet activation in diabetics, favoring ischemic stroke (40). Furthermore, circulating levels of miR-223 were found to independently predict the response to clopidogrel treatment, which shows potential use of this miRNA in the assessment of efficacy of antiplatelet therapy (35).

Altogether, miR-223’s diverse and complex regulatory functions suggest this miRNA might be used as a potential biomarker of platelet activation, a surrogate marker of antiplatelet treatment efficacy, as well as predictor for the risk of cardiovascular death and a tool in T2DM diagnosis.

## miR-126

Another important miRNA associated with platelet function, miR-126, is located on human chromosome 9 (5). Both strands of miR-126: miR-126-3p and miR-126-5p, are biologically active (41). miR-126 belongs to the most abundantly expressed miRNAs in endothelial cells being responsible for vascular development, integrity, and response to hemodynamic stress (25).

Although miR-126 has been linked to angiogenesis and to the development of CVD in several independent reports, few studies focused on its role in platelet activation (18, 25, 42). Willeit et al. revealed that platelet inhibition through administration of antiplatelet drugs [10 mg prasugrel or 10 mg prasugrel with 75 mg acetylsalicylic acid (ASA)] resulted in reduction of miR-126 levels (1). Moreover, de Boer et al. confirmed the correlation between the concentration of miR-126 in plasma and platelet activation *in vivo*. In fact, ASA administration resulted in reduction of circulating platelet-derived miR-126 in patients with T2DM (42). Kaudewitz and coworkers independently confirmed that alterations in miR-126 affect platelet reactivity (18). These results underline the key role of miR-126 in platelet activation.

Furthermore, Zampetaki et al. suggested that alterations in circulating miR-126 levels have a diagnostic and prognostic value as a biomarker for endothelial dysfunction in T2DM (43). In another study by Olivieri et al., the expression of miR-126-3p in healthy controls was markedly increasing with their age, what was paralleled by a raise of intra/extracellular miR-126-3p in senescent *in vitro* cultured human endothelial cells (HUVECs) (44). Interestingly, such age-related differences in miR-126-3p plasma levels were not observed in T2DM patients. On the other hand, when compared with age-matched controls or T2DM patients with appropriate glycemia, miR-126-3p levels were lower in T2DM patients with poor glycemic control. In an *in vitro* model, miR-126-3p expression in HUVECs cultured in high glucose medium was significantly lower than in HUVECs exposed to low glucose concentration. These results indicate that miR-126-3p might be evaluated as a biomarker of physiological senescence of endothelial cells in patients with appropriate glycemia level, but also for impaired survival of endothelial senescent cells exposed to high glucose levels in T2DM patients (44). Stratz et al. assessed platelet miRNA profiles in a cohort of 60 patients, including clinically stable diabetic, and non-diabetic patients, and no significant differences in plasma miR-126-3p between diabetic and non-diabetic patients were noted (45). Zampetaki et al. sought to evaluate miRNA profiles in diabetic subjects and a potential association between miRNA expression and MI. High glucose concentration resulted in significantly reduced miR-126 amount in endothelial apoptotic bodies, followed by reduced miR-126 plasma level. Since miR-126 facilitates VEGF signaling by repressing SPRED1 and PIK3R2/p85-β, it has been suggested that low plasma miR-126 levels might have an impact on VEGF resistance and endothelial dysfunction in patients with T2DM (25). Furthermore, moderate decrease in miR-126 levels in normal glucose, impaired fasting glucose/impaired glucose tolerance and T2DM was observed (43). Hence, the decreased level of miRNAs that are highly expressed in platelets, such as miR-126, may reflect platelet dysfunction in the diabetic population.

miR-126 has been proposed as a candidate biomarker of cardiac diseases, as it was positively correlated with incident MI (38). Prognostic values of several miRNAs (miR-126, miR-21, miR-130, miR-222, miR-20a, miR-let7d, miR-27a, miR-92a, miR-17, miR-199a) for the occurrence of cardiovascular events in patients with stable coronary disease were reported in later studies (46, 47). In particular, Jansen et al. reported no significant association between cardiovascular events (median follow-up period was 6.1 years) and plasma levels of the selected miRNAs. Nonetheless, among the miRNAs tested, increased levels of miR-126 in circulating microvesicles were reported to correlate with lower predisposition to major adverse events in patients with stable coronary artery disease (CAD) (46). The positive effect of miR-126 on cardiovascular system was established by Harris and colleagues, who proved that the level of endogenous miR-126 is negatively associated with VCAM-1 expression. As a result, alterations in miR-126 expression control vascular inflammation by influencing leukocyte adhesion to endothelium (48). de Rosa et al., on the other hand, measured miR-126 concentration in patients undergoing coronary angiography who were separated into 3 groups depending on evidence of coronary artery disease and troponin (hsTNT) levels. The samples were obtained from both coronary venous sinus and aorta. They revealed that miR-126 level in aorta was positively correlated with hsTNT concentration. Interestingly, only in patients with acute coronary syndrome miR-126 concentration in CVS was lower than in the aorta, suggesting consumption of endothelial miR-126 during transcoronary passage (49).

However, the administration of antiplatelet drugs should be considered when using circulating miR-126 and possibly other platelet-derived miRNAs as diagnostic biomarker for CVD (42). Carino et al. showed that the change of antiplatelet treatment also influences circulating levels of miR-126. In their study, the circulating levels of miR-126 were significantly reduced after switching from DAPT with ASA and clopidogrel to ticagrelor (50).

In line with these results, miR-126 was reported to correlate with elevated risk for MI (38). Yu et al. suggested that plasma miR-126 may serve as a future marker prognostic of major adverse cardiac events in patients after percutaneous coronary intervention (PCI) in a study conducted on 491 Han Chinese individuals who had received PCI and DAPT (51). On the contrary, Schulte et al. reported in their work that miR-126 expression did not demonstrate any significant prognostic value, neither in the overall group, nor in ACS or the stable CAD group (37). Given the diverging results, it is mandatory to perform further studies in order to validate the usefulness of miR-126 in assessment of cardiovascular death risk stratification.

Summing up the above reported results, accumulating evidence indicates that miR-126 might be used as an innovative biomarker and potential novel therapeutic target through its roles in maintaining endothelial homeostasis. However, to better evaluate the potential role of miRNA-based therapy more studies will be required to investigate the intricate interactions between this miRNA and its target genes in T2DM, CAD, and other CVD.

## miR-197

miR-197, found on human chromosome 1, is among the most highly expressed miRNAs in platelets (1, 38, 52). However, its role in platelet activation is not fully defined, yet. In addition, it has been established that miR-197 might contribute to dyslipidemia in metabolic syndrome, hence leading to the progression of CVD (53).

Schulte and colleagues evaluated the prognostic aspect of serum-derived circulating miR-197 in a large population of patients with CAD (*n* = 873). Cardiovascular death was seen in 2.1% of the patient cohort over a follow-up period of 4 years and baseline levels of three miRNAs, one of them being miR-197, were more elevated in individuals with future cardiovascular death relative to event-free subjects. According to their results, miR-197 could serve as a prognostic biomarker of future cardiovascular death (37). Also Zampetaki and colleagues investigated the link between baseline miRNAs levels and incident MI in the Bruneck cohort and revealed that miR-197 negatively correlated with disease risk [multivariable hazard ratio: 0.47 (95% CI: 0.29–0.75), *P* = 0.002, and 0.56 (95% CI: 0.32–0.96), *P* = 0.036] (38). The team found that investigated miRNAs were predominantly expressed in platelets. These findings suggest that the observed loss of numerous miRNAs, including miR-197, may indicate abnormal platelet function in T2DM population (38). In another study, lower plasma levels of miR-197 were revealed in subjects with manifest T2DM (25). Whereas this study supported the concept of plasma miRNAs being abnormally regulated in T2DM, the underlying mechanism has not been exhaustively clarified. The results suggest that plasma miRNAs, including miR-197, might be a useful tool to predict both cardiovascular death and T2DM. However, these results require an independent validation in larger groups of CAD patients and prediabetes before more definitive comparisons with other standard risk factors can be made.

Human platelets harbor a diverse and complex miRNA repertoire. Beside the abovementioned three most known miRNAs, some other types count toward the most highly expressed miRNAs in human platelets and have a capacity to influence platelet function (54).

## miR-191

miR-191 is located on human chromosome 3 and expressed both in platelets and endothelial cells (55, 56). It was one among 377 miRNAs profiled in a seminal study performed by Willeit et al., which showed remarkably higher levels of miR-191 in serum than plasma. Interestingly, plasma levels of platelet miR-191 were also found to be decreased on platelet inhibition with prasugrel and ASA (1).

In another study performed in 39 patients, Hsu et al. unveiled that miR-191-5, as well as miR-486-3p were markedly reduced in the sera of patients with acute myocardial infarction (AMI) (57), suggesting that they could be potential diagnostic biomarkers for ST segment elevation myocardial infarction (STEMI) (57). Also, Li et al. assessed the expression of miRNA in a cohort of AMI patients and healthy subjects to establish whether plasma levels of miR-26a, miR-191, and miR-208b could be clinically useful biomarkers of AMI. The study, which included 87 AMI patients and 87 healthy individuals, revealed that miR-191 and miR-26a were decreased in AMI relative to healthy subjects. A good diagnostic performance was found for miR-191 (AUC = 0.669; 95% CI: 0.589–0.749; *P* < 0.001) (58). On the other hand, Kakimoto et al. analyzed the possible application of miRNA quantification during postmortem examination of AMI patients. Among 55 samples of cardiac tissue that were collected and examined, miR-191 and miR-26b showed sufficient stability after death and long-lasting fixation, to be considered as candidate biomarkers in this setting (59).

In a population of T2DM patients, Dangwal et al. reported a three- to sixfold decrease in plasma levels of circulating miR-191 in diabetic versus healthy controls. miR-191 was correlated with cytokine levels and C-reactive protein (*r* = 0.333; *P* = 0.009) in T2DM subjects and indeed, pro-inflammatory stress caused higher secretion of endothelial- or platelet-derived miRNA (56).

Currently, cardiac troponins and creatinine kinase-MB are the most common biomarkers for MI. Nevertheless, these are biomarkers of myocardial necrosis, while miRNAs could provide broader information about a wider range of biological processes, potentially allowing an earlier diagnosis. It is therefore of paramount importance to promote further research to increase the current efficiency of detection methods for miRNAs (60). Although the usefulness of miR-191 appears to be modest, current data suggest probable involvement of platelet-secreted miRNAs in the plasma pool of T2DM patients (56).

## miR-21

The miR-21-5p is located on human chromosome 17 (5, 61). It is highly expressed in cardiovascular cells, such as vascular smooth muscle cells (VSMCs), endothelial cells (ECs), cardiac fibroblasts (CF), and cardiomyocytes (CMC), as well as in platelets (38, 62).

In order to assess the potential application of atherosclerosis-related miRNAs (miR-361-5p, miR-21-5p, and miR-519e-5p) in the diagnosis of AMI, Wang et al. evaluated the expressions of circulating miRNAs in individuals with AMI. Plasma level of miR-21 in this cohort was significantly elevated relative to healthy volunteers without a history of CAD. Interestingly, circulating miR-21 exhibited similar trend to plasma cardiac troponin I in the early phase of AMI with both of them achieving the peak concentration at 4 h after initial time, and declining in the subsequent hours. Clinical application of miR-21 for diagnosing and monitoring AMI were also assessed. As miRNA quantitative analysis demonstrated elevated expression of miR-21 not only in patients with AMI but also in patients with stroke or pulmonary embolism, it could lack sufficient specificity to be exploited as a disease biomarker (63). On the other hand, Zhang et al. found that plasma levels of miR-21 were significantly higher in patients with AMI or angina compared with controls. They also found a significant correlation between miR-21 and clinically established markers, including cTnI, creatine kinase, and creatine kinase (*P* < 0.001) reinforcing the concept of the application of circulating miRNAs in disease diagnosis and prognosis (64, 65).

In another study conducted by Cengiz et al., miR-21 was investigated as a potential contributor to subclinical atherosclerosis among hypertensive patients. In a cohort of 28 hypertensive subjects and an equal number of healthy volunteers, plasma miR-21 expression was markedly elevated in the hypertension group *vs*. the control group. Furthermore, miR-21 levels were positively associated with both systolic and diastolic blood pressure, suggesting that it may play a role in initial stages of atherosclerosis in patients with hypertension (66).

On the contrary to cardiac disorders, in which miRNA-21 is the most abundantly expressed miRNA, in patients with T2DM, the plasma level of miR-21 was found to be reduced (5, 25). The same trend was confirmed by Olivieri et al. who carried out a study on 193 T2DM patients and 107 healthy control subjects; however, they also established that expression of miR-21-5p was higher in patients with T2DM and major cardiovascular events as compared to other T2DM patients (61). On the contrary to cardiac disorders, in which miRNA-21 is the most abundantly expressed miRNA, in patients with T2DM the plasma level of miR-21 was lower in patients with T2DM compared to controls (5, 25).

In summary, miR-21 is a promising biomarker of ischemic cardiovascular diseases, as well as T2DM. Nonetheless, further studies should be designed to disentangle the conflicting results available to date in T2DM.

## miR-150

Despite the fact that this miRNA has not been associated to prognosis in T2DM patients, miR-150 is worth mentioning in this review, as it has an impact on platelet maturation and function. miR-150 is found on human chromosome 19 and it is known to be a key modulator of platelet production and activation (50, 67). It is highly expressed in leukocytes and monocytes, where it targets c-Myb, a transcription factor associated with cell proliferation, lineage commitment, and migration (34).

It was discovered that miR-150 levels were upregulated as megakaryocyte-erythrocyte progenitors (MEPs) differentiated toward the megakaryocyte lineage (68). The contribution of miR-150 to megakaryocyte differentiation was also confirmed by Barroga et al. In fact, they found that thrombopoietin increases the expression of miR-150 (69). Nevertheless, miR-150 was reported to influence not only platelet production, but also their activation. Willeit et al. revealed that more potent platelet inhibition in healthy subjects resulted in lower levels of miR-150 (1). Correlation between miR-150 level and platelet activation is further and independently supported by experimental result from our group (50). In fact, similarly to miR-126, the circulating levels of miR-150 were significantly lower after the switch from clopidogrel to ticagrelor (50). Furthermore, Yu and colleagues found that miR-150 is upregulated in platelets by apheresis (70).

The diagnosis of unstable angina (UA) based on time-specific biomarker remains a major clinical challenge. Zeller et al. performed a study aiming to assess clinical utility of miRNAs as a new tool in patients with UA. By using a three-phased profiling-replication-validation model, they found eight miRNAs which could be used clinically in the early diagnosis of UA. Among selected miRNAs, miR-186 demonstrated the greatest correlation with UA. However, the triple-miRNA combination of miR-150, miR-132, and miR-186 was shown to be of highest diagnostic accuracy indicating that a multi-miRNA approach is more reliable than single miRNAs (71). In another study conducted by Zhang et al., the level of circulating miR-486 and miR-150 in patients with AMI and their prospective role as biomarkers for AMI were investigated. The study included 65 STEMI patients, 45 non-ST-segment elevation myocardial infarction (NSTEMI), and 110 healthy subjects. According to PCR results, plasma miR-486 and miR-150 were significantly higher in all AMI (both STEMI and NSTEMI) subjects. Moreover, miR-150 was considerably overexpressed in the initial stage of AMI in patients, relative to healthy subjects (*P* < 0.001). An evident difference was reported between the levels of plasma miR-150 and miR-486 between STEMI and NSTEMI subjects, suggesting that miR-150 could be helpful to distinguish NSTEMI patients from healthy volunteers (72). Recently, also Karakas et al. evaluated the potential role of eight miRNAs (miR-19a, miR-19b, miR-132, miR-140-3p, miR-142-5p, miR-186, miR-150, miR-210) as prognostic biomarkers for CVD in a large population of 1112 CAD patients (430 ACS patients, 682 stable CAD patients). They found that the majority of miRNAs were predictive of cardiovascular death in ACS patients during a follow-up of 4 years (73). Furthermore, Goren et al. reported that miR-150 levels correlate with platelet count in heart failure (HF) patients and its expression levels were 3.5-fold lower in subjects with HF and atrial fibrillation (AF) compared to HF subjects without AF (74).

On the basis of the above reported evidence, it can be hypothesized that miR-150 levels in peripheral blood could be used to predict mortality in secondary prevention settings.

## miR-155

miR-155 is located on human chromosome 21. Its platelet content is significantly reduced in diabetics (16). miR-155 is enriched in inflammatory microvesicles in patients with cardiovascular or dysmetabolic diseases. Nevertheless, detailed information on its function in cardiovascular pathophysiology is lacking, to date. Therefore, its potential role as biomarker needs further investigation (12, 75).

## miR-140

So far, data on the association between miR-140 and platelet reactivity or function are limited. This miRNA is located on human chromosome 16 (76). Fejes et al. aimed to investigate the concentration of circulating platelet miR-126, miR-140, miR-223, and miR-26b that might be modified by their target mRNAs in T2DM, in a study involving 70 subjects. It was found that miR-140, miR-223, miR-126, and miR-26b are reduced both in platelets and megakaryocytes of T2DM patients, resulting in upregulation of P2RY12 and P-selectin mRNAs. This might in turn lead to abnormal platetet function (34). Karakas et al., on the other hand, suggested that miR-140-3p is a promising prognostic marker in CAD patients (73).

## miR-96

Circulating levels of miR-96, located on human chromosome 7, are associated with platelet function, both in normally responsive patients and in the setting of platelet hyperreactivity (50, 77, 78). Those findings are particularly interesting, as it was reported that upregulation of miR-96 parallels hyperexpression of vesicle-associated membrane protein 8 (VAMP8)/endobrevin, a known v-SNARE involved in platelet degranulation (78). Noteworthy, VAMP8 itself is a target of miR-96 (78).

## miR-98

miR-98 that is located on human chromosome X, belongs to miRNAs, which have been scarcely described and analyzed in respect to their role in platelet function, to date (79). However, Osman et al. demonstrated that miR-98 was among the miRNAs (miR-15 a, miR-98, miR-339-3 p, miR-361-3 p, miR-365, and miR-495) that are deregulated upon platelet activation (*P* ≤ 0.001). These data provide valuable information on potential miRNA target pathways in platelets, even though further studies are required to precisely evaluate their actual usefulness as biomarkers of CVD (80).

## Future Perspectives of miRNA

Circulating miRNAs are stable in both plasma and serum and were shown to have prognostic values for CVD (81–84). Thus far, three main pools of plasma circulating miRNAs have been verified: protein-bound, high density lipoprotein (HDL)-associated, and microvesicle (MV)/exosome-associated miRNAs (85). It has been described that among these three pools, a significant amount of plasma miRNAs is associated to microvesicles (86). However, Arroyo et al. reported that miRNA can be also found in a vesicle-free form bound to RNA-binding proteins, including nucleophosmin and Argonaute protein 2 (83, 87, 88).

Recent data demonstrate that age and gender play a role in microRNA–RNA interactions in platelets. Given that these demographic variables have a considerable impact on cardiovascular and diabetes prevalence, morbidity, and mortality, the link between age- and gender-related differences in miRNA expression could further enhance the prognostic value of miRNA. Simon et al. evaluated mRNA and miRNA levels in platelets from 84 white and 70 black healthy subjects. Out of 5,911 mapped mRNAs and 181 miRNAs that were expressed and validated in a separate cohort, 129 mRNAs and 15 miRNAs were differentially expressed by age, and 54 mRNAs and 9 miRNAs by gender. The results suggest that miRNAs modulate mRNA levels on aging and between genders, and hence these variables could be incorporated into a predictive model for platelet reactivity biomarker (89).

As reviewed above, platelets harbor large amounts of miRNAs. Consequently, they are the major source for circulating miRNAs, with a relevant regulatory potential on cardiovascular pathophysiology. Platelet-derived miRNAs could be exploited as useful biomarkers for clinical use (90). Nevertheless, several issues should be solved to enable effective application of circulating platelet-derived miRNAs as disease biomarkers in the clinical setting. In fact, several innovations are being put forward in the diagnostic methodology. Among the others, fast PCR-based techniques merit mention. In addition, the use of primers bound on multi-well plates also represents a valuable progress. Furthermore, microfluidic systems have been also developed to filter blood samples before analysis, allowing fast detection with no need for time-consuming centrifugations. These techniques can also be associated to advanced detection methods, such as microarrays. Using an alternative approach, enzyme-linked assays are being developed, to allow miRNAs measurement through direct hybridization. Among the most promising, the use of nanosensors or nanowires will be an active field of technological development over the next years. These latter, combined with microfluidic filtering devices, will allow the development of reliable and efficient, label-free detection methods (91).

Current evidence on the pathophysiological role played by miRNAs in this specific topic is still not sufficient to support their use as therapeutic targets using currently available technologies. For example, many of the miRNAs discussed in this review are downregulated in the diseased state, while current therapeutic strategies in other fields are mostly based on inhibition of abnormally elevated miRNAs. In fact, therapeutic increase of miRNAs *in vivo* is more challenging with the currently available bio-tech armamentarium. More studies should be designed with an aim to better understand the precise involvement of miRNAs in this specific pathophysiological context, to allow the identification of specific therapeutic targets.

## Conclusion

Upon activation, platelets secrete microvesicles that carry large amounts of growth factors, as well as various proteins which might exert extracellular effects. Recent studies have indicated that PMVs may deliver platelet miRNA to a specific site in the cardiovascular system (10). The platelet-derived miRNAs that have the highest association with CVD are miR-223 and miR-126. miR-223 regulates erythrocyte membrane protein band 4.1 like 3 (*EPB41L3*) gene, known to be linked to atherosclerosis, while miR-126 regulates a gene that is strongly linked to endothelial dysfunction and atherosclerosis as vascular cell adhesion molecule 1 (VCAM-1), therefore indicating that platelet-derived miRNAs have an impact on the regulation of key genes associated with CVD (5). In our previous studies, in a cohort of T2DM patients, we found that platelet reactivity could be related to a number of clinical factors, biochemical variables, and genetic polymorphisms (92–99). Moreover, we found that genetic polymorphisms within genes related to platelet reactivity could be also a useful prognostic tool (12). Similarly, to gene polymorphisms, miRNA profiling may expose inter-individual differentiation in platelet reactivity, disease susceptibility, or response to therapy.

Results of the studies presented in this review should be interpreted with consciousness. The discrepancy of results might stem from demographic differences between populations, heterogeneity of populations, various cohort sizes and study designs. It is necessary to conduct further studies to validate the current hypotheses and closely determine the association between various miRNA and platelet reactivity, as well as their contribution to cardiovascular diseases development.

Altogether, miR-223, miR-126, miR-197, miR-24, and miR-21 were found to be the most abundant miRNAs in human platelets and PMVs and may contribute to the plasma miRNA pool (1, 43, 100). Mounting evidence suggests that platelet miRNAs could be exploited as biomarkers in inflammatory diseases, including T2DM and CVD, as they influence a broad spectrum of cell mechanisms and functions. Given the abundance of platelets in the blood and their substantial contribution to the circulating miRNA pool, these cells could serve as the main source for this class of biomarkers. Presently, few biomarkers could be applied clinically to verify subjects at higher risk of development of acute presentation of CVD. Despite use of classical cardiovascular risk factor and the development of numerous risk stratification models, a significant proportion of cardiovascular risk still eludes currently available risk stratification strategies (101). This residual risk is partly related to genetic variability, partly associated to environmental factors that are not captured by available risk models (102, 103). Hence, miRNAs could be useful epigenetic biomarkers, while able to sense both genetic and environmental risk components.

## Author Contributions

JP, KP, SR, and MP: substantial contribution to concept and design and critical writing or revising the intellectual content. SR and MP: edition of the manuscript and supervision of the work. CE: valuable contribution to graphic design. CI: critical revision of the article. JP, KP, SR, MP, CE, CI, ADJ and LM: collection, analysis, and interpretation of data; verification of analytical methods; and final approval of the version to be published. All authors agreed to be accountable for all aspects of the work in ensuring that questions related to the accuracy or integrity of any part of the work are appropriately investigated and resolved.

## Conflict of Interest Statement

The authors declare that the research was conducted in the absence of any commercial or financial relationships. No conflict of interest exists.
