# Supplementary figures and images for: The Potential Role of Platelet-Related microRNAs in the Development of Cardiovascular Events in High-Risk Populations, Including Diabetic Patients: A Review
Source: Front Endocrinol (Lausanne). 2018 Mar 20;9:74. doi: 10.3389/fendo.2018.00074 (PMC5869202; doi:10.3389/fendo.2018.00074)

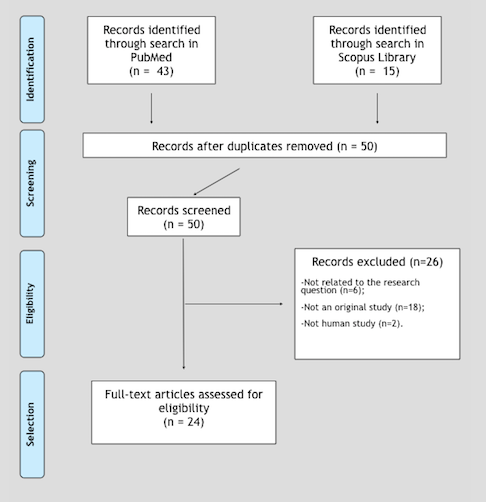

Supplement: Supplementary file 1 [file image_1.tiff]
