# Supplementary material for: The Potential Role of Platelet-Related microRNAs in the Development of Cardiovascular Events in High-Risk Populations, Including Diabetic Patients: A Review
Source: Front Endocrinol (Lausanne). 2018 Mar 20;9:74. doi: 10.3389/fendo.2018.00074 (PMC5869202; doi:10.3389/fendo.2018.00074)
Supplement: Supplementary file 2 [file table_1.docx]

| **miR** | **Aim of the study** | **Clinical endpoint** | **Sample size** | **Control cohort** | **HR value** | **Reference** |
| --- | --- | --- | --- | --- | --- | --- |
| MiR-223 | Prognostic value in patients with documented CAD | Cardiovascular death | 873 | - | 2.23 | [37] |
|  | Association with incident MI | Fatal and nonfatal MI | 820 | - | 0.47 | [38] |
|  | Prognostic value in the prediction of cardiovascular outcome and death | All-cause mortality | 178 | 129 | 0.30 | [39] |
| MiR-126 | Association with incident MI | Fatal and nonfatal MI | 820 | - | 2.69 | [38] |
|  | Prognostic value for the occurrence of CV events | Occurrence of a first major adverse CV event | 181 | - | 0.485 | [46] |
| MiR-197 | Prognostic value in patients with documented CAD | Cardiovascular death | 873 | - | 1.77 | [37] |
|  | Association with incident MI | Fatal and nonfatal MI | 820 | - | 0.56 | [38] |
| MiR-191 | Alteration of miRNA in STEMI | STEMI | 31 | 31 | - | [57] |
|  | miRNA expression in AMI | AMI | 87 | 87 | - | [58] |
| MiR-21 | miRNA expression in AMI | AMI/ stroke/ pulmonary embolism | 43 | 40 | - | [63] |
|  | Potential role in diagnosis of AMI | AMI | 56 | 10 | - | [64] |
|  | Potential contributor tion to subclinical atherosclerosis among hypertensive patients | Hypertension | 28 | 28 | - | [66] |
| MiR-150 | Association with unstable angina pectoris | Unstable angina pectoris | 10 | 20 | - | [71] |
|  | Association with AMI | AMI | 110 | 110 | - | [72] |
|  | Association with cardiovascular diseases | Cardiovascular death | 1112 | - | 2.14 | [73] |
|  | Association with presence of AF | Heart failure with AF | 41 | 35 | - | [74] |
| MiR-140 | Association with cardiovascular diseases | Cardiovascular death | 1112 | - | 2.88 | [73] |

Table 1. Summary of studies on cardiovascular diseases included in the review.

HR – hazard ratio, CAD – , MI – myocardial infarction, CV- cardiovascular, STEMI - ST-segment elevation myocardial infarction, AMI – acute myocardial infarction, AF – atrial fibrillation
